# Proceedings of the Lippe Society

**Published:** 1889-02

**Authors:** Geo. H. Clark

**Affiliations:** Secretary


### PROCEEDINGS OF THE LIPPE SOCIETY.

The 128th meeting of the Lippe Society was held on Tuesday evening, January 8th. After the minutes of the last meeting were read and approved, an election for officers was held. Dr. C. Carleton Smith was elected President; Dr. F. Powel, Vice-President, and Dr. George H. Clark, Secretary-Treasurer. Dr. Preston, commenting on metastasis of disease, said, a few years ago a lady came to him with an attack of sciatica. The pains were of an excruciating character, commencing in hip, going half-way down the thigh, skipped the knee, and then went to the ankle and foot. The leg was shortened to the extent of six inches, due to muscular contraction. She had been under the treatment of an allopathist for a number of weeks without relief. After a few days of study, Dr. Preston gave three doses of Kali-hyd.$^{10m}$, as he learned that the same allopath had treated her in infancy for an eruption, and had suppressed it with red precipitate. In one week after taking the Kali-hyd. she was well. The point of the case is, said Dr. Preston, this: When I left her, I advised her never to have an external application made for any disease. Marrying a few years after, and after her first delivery, she was subject to leucorrhœa. She was attended by her old allopath. He gave her an injection of Alum and stopped the leucorrhœa. In a few months she was an inmate of an insane asylum. While there the leucorrhœa reappeared, and the insanity disappeared. She became pregnant the second time, went through the same routine, and was again sent to the asylum; the leucorrhœa again appeared, and her mind became clear. This case, and all similar cases go to show the bad effects of suppressed disease manifestations.

Dr. Lee then proposed, and the proposition was accepted, that, at each meeting, instead of having two or more subjects for discussion, one member be appointed to prepare a paper on some special disease, and one member appointed to lead in discussing the subject.

Dr. Farley then presented the following case, having the patient present:

Mr. I. T. E., forty-eight years of age. Bilio-nervous temperament. Has suffered for five or six years with recurring attacks of severe pain in abdomen, epigastrium, and chest. Seemingly indicated remedies have given him relief for periods varying from two or three days to as many months. Pain at present is located at about centre of left hypochondriac region, extending downward and posteriorly to lumbar region; is stabbing and burning in character, and sometimes a dull pain gradually expanding and as gradually retracting, and again a "pressing-out pain," as if a blunt instrument were forcing itself to surface, anteriorly, in location named. Occasionally a painful feeling, as if an apple-core had been swallowed, from fauces to stomach. On two occasions during the past year, has passed renal gravel with much suffering, severe, but quickly relieved by indicated remedy. First from right kidney, and later from left. Suffers from attacks of "sick headache," preceded by blindness, that are speedily relieved by Iris-v. The attacks of headache are becoming much less severe and frequent. *Pains are in* P. M., *about four o'clock; ten* P. M. *if not in bed asleep, and when stomach is empty;* SEVERE FROM EATING FULL MEAL.

Last remedy given was Phos. Remedies recommended for study are Phytolac., Hepar, Phos., Lycopo.

Dr. Smith thought Phytolacca approached the case. The characteristics of that remedy are burning pains; red-hot pains, especially in the throat, and sensation of apple-core in throat.

Dr. James—There are only three remedies mentioned by Bœnninghausen having relief after eating enough: *Ars.*, Iod., PHOS.

Dr. Powel—Hepar always better after a hearty meal. And Anacardium, said Dr. Farley.

Dr. Lee advised the continuation of Phosphorus in a higher potency.

Dr. Powel once cured severe pains in epigastrium, where patient had to hurry to get his meals, in order to relieve the pain; particularly dinner and supper. Graphites was the remedy.

Dr. James said that Dr. Lippe had given him that symptom some years ago as belonging prominently to Graphites.

Dr. Lee then read a paper on "Acute and Chronic Tonsillitis." (See page 77.) Dr. Preston made a motion, which was adopted, that Dr. Lee be requested to prepare a repertory of the remedies mentioned in his paper.

After several remedies were suggested in addition to those named in the paper, Dr. Preston related a case of malignant diphtheria, in which the child was unable to swallow anything. Being unable to speak, she motioned that she wanted ice-cream, which, on being given, it was found that she could not swallow a small particle, but larger pieces were readily swallowed. Although moribund at the time, Lach. brought her up.

Dr. Farley then asked for a remedy with the symptom, must stand up to eat. A case of angina pectoris. Alumina was suggested for study.

Dr. Powel asked for remedy for nervous tremor and twitching about right eye and right cheek. Dr. Smith named Mag-phos. Dr. Powel had given Agaricus without effect.

Dr. Lee then related a case of a young girl who has swelling on neck; sebaceous tumor on head; much dandruff, and hair falling out. Menses too early and scanty. If menses first appear in the morning, she has three or four normal stools; if the flow comes on in the evening or night, she has no trouble. Dr. Preston suggested Bovista.

After deciding upon diphtheria as the subject for next meeting, the President appointed Dr. Still, of Norristown, to prepare the paper, and Dr. Powel to lead the discussion. The Society then adjourned.

GEO. H. CLARK, *Secretary*.

